# Connecting Sarcomere Protein Mutations to Pathogenesis in Cardiomyopathies: The Development of “Disease in a Dish” Models

**DOI:** 10.3389/fphys.2016.00566

**Published:** 2016-11-22

**Authors:** Rebecca Zaunbrecher, Michael Regnier

**Affiliations:** ^1^Department of Bioengineering, University of WashingtonSeattle, WA, USA; ^2^Center for Cardiovascular BiologySeattle, WA, USA; ^3^Institute for Stem Cell and Regenerative Medicine, University of WashingtonSeattle, WA, USA

**Keywords:** stem cell derived cardiomyocytes, gene editing, cardiomyopathies, contraction, methods development

## Abstract

Recent technological and protocol developments have greatly increased the ability to utilize stem cells transformed into cardiomyocytes as models to study human heart muscle development and how this is affected by disease associated mutations in a variety of sarcomere proteins. In this perspective we provide an overview of these emerging technologies and how they are being used to create better models of “disease in a dish” for both research and screening assays. We also consider the value of these assays as models to explore the seminal processes in initiation of the disease development and the possibility of early interventions.

It has now been over 35 years since the first report of a genetic linkage between a sarcomere protein mutation (or variant) with a disease phenotype (Geisterfer-Lowrance et al., 1990). Since then well over 1000 genetic variants of sarcomere proteins have been reported as associated with diseases such as hypertrophic, dilated, or restrictive cardiomyopathy, though many fewer have been confirmed as having causative roles (Seidman and Seidman, 2011; Haas et al., 2015). The vast majority of research to understand the phenotypic consequences of these genetic variants has been done using post-natal animals, cell culture, and recombinant protein models. Thus, much has been learned about later stages of the disease process, likely after multiple compensatory processes have been invoked and often when hearts are in failure. However, there is growing consensus that to find effective treatments for these familial diseases it is important to understand the role of these mutations in earlier stages in disease progression, before clinical signs manifest and perhaps at the earliest stages of human development.

The development of technologies to derive human pluripotent stem cells and differentiate them into cardiomyocytes has provided a model system in which to study these crucial early stages of development. Beginning with the derivation of the first human embryonic stem cell line in 1998 (Thomson et al., 1998) and continuing with the derivation of human induced pluripotent stem cells (hiPSCs) in 2007 (Takahashi and Yamanaka, 2006), it has been theoretically possible to generate any tissue in the lab. However, early differentiation protocols for cardiomyocytes depended upon the spontaneous formation of embryoid bodies and resulted in low yields of cardiomyocytes (~1–5%; Kehat et al., 2001). Better understanding of pathways involved in heart development *in vivo* has since been leveraged to develop more efficient differentiation protocols and it is now possible to achieve >90% pure cardiomyocyte populations using multiple methods (Spater et al., 2014). Additionally, these technologies in reprogramming and directed differentiation have made it possible to generate cell lines and large numbers of cardiomyocytes from patient samples and have laid the foundation for hiPSC-based cardiac disease modeling.

A series of technological advances in genomic engineering have also advanced the ability to model cardiomyopathies with hiPSC-derived cardiomyocytes (hiPSC-CMs). Until recently, cell lines have largely been generated using samples from patients who both (1) carry a known or suspected pathogenic mutation and (2) clinically present with cardiomyopathy. A major benefit of this approach is that the resulting cell line will carry both the mutation of interest as well as any undefined or unknown genetic modifiers that may be necessary for presentation of the disease phenotype. This enhances the likelihood that a phenotype will emerge *in vitro*. However, it can often be difficult to establish appropriate controls for patient-derived cell lines. Even control cell lines established from healthy close relatives can have significant genetic variation compared to the cardiomyopathy line, and when relatives' samples are unavailable often completely unrelated wildtype cell lines are used (Siu et al., 2012; Lin et al., 2015). Nevertheless, this has been used to successfully study both hypertrophic (Lan et al., 2013) and dilated (Sun et al., 2012; Wu et al., 2015) cardiomyopathy and gain mechanistic insights into the pathogenesis of these mutations.

Breakthroughs in genome engineering technologies, notably the development of the CRISPR/Cas9 system for use in mammalian systems, currently allow for the generation of isogenic control lines in hiPSC-CM modeling. Using a 20-base pair single guide RNA, the CRISPR/Cas9 system can create double-stranded breaks at nearly any location in the genome. This allows for the straightforward generation of random mutations using non-homologous end joining (NHEJ), as well as specific base pair changes at lower efficiencies by supplying a template for homology-directed repair (HDR; Ran et al., 2013). More recent advances have focused on improving the efficiency of HDR (Chu et al., 2015; Yu et al., 2015) and increasing the target range of CRISPR systems by mutating the commonly used Cas9 nuclease (Kleinstiver et al., 2015a) and deriving nucleases from different species of bacteria (Kleinstiver et al., 2015b).

Two general approaches are available for using genome engineering in hiPSC-CM modeling. In the first, mutations can be specifically engineered into a healthy, wildtype hiPSC line. A significant benefit to this approach is that many different mutations can be tested on the same genetic background, allowing for a rigorous comparison. Particularly for non-sense mutations, this can be done in a relatively high-throughput manner through the use of NHEJ. Alternatively, mutations in a patient-derived line can be corrected using HDR. However, this is often technically more problematic, and it can be difficult to discern if performance returns to healthy, wildtype levels. Although these genome engineering technologies are still relatively new, it is clear that controls generated using these techniques are more accurate than cell lines created from unaffected relatives or unrelated individuals. In the future, isogenic controls should be considered a standard in hiPSC-CM disease modeling.

There are numerous technologies for phenotyping hiPSC-CMs. Of particular interest in many cardiomyopathies are mechanical function measurements of hiPSC-CMs, which can be acquired on single cells or in the context of a multicellular tissue engineered system. Current assays allow for highly sensitive measurements of both force production and kinetics of hiPSC-CM contraction and relaxation, and these measurements have been successfully used to characterize the phenotype of several hiPSC-CM cardiomyopathy models (Sun et al., 2012; Hinson et al., 2015). Recent reviews provide comprehensive coverage of both single cell measurement systems (Polacheck and Chen, 2016) and multicellular tissue engineering approaches (Tzatzalos et al., 2016). Of note, a method has recently been developed to mature hiPSC-CMs sufficiently to harvest isolated myofibrils for mechanics measurements to determine how sarcomeric mutations directly affect the organelle in which they are located (Pioner et al., 2016).

With the addition of this assay, it is now possible to characterize the functional properties of hiPSC-CMs on subcellular, cellular, and multicellular levels, a crucial set of tools for cardiomyopathy disease modeling. Studying the effects of disease-associated mutations at each level of organization provides insight into distinct aspects of cardiomyocyte function and how they are affected by the mutation of interest. Myofibril mechanics measurements assess the contractile ability of the hiPSC-CMs independent of the influences of the cell's Ca^2+^ handling properties or intracellular signaling pathways. Single cell measurements assess the function of myofibrils in the context of whole-cell function, but without the influence of cell-cell communication. Finally, multicellular tissue constructs provide insight into the effects of cellular junctions and environmental cues from extracellular matrix, and is currently the most physiological setting in which to study hiPSC-CMs *in vitro*. By performing these multiscale analyses in an *in vitro* setting, where the internal and external environments can be manipulated, it is now possible to study how molecular level changes in myofilament protein structure and function (with mutations) affect contractile fibrils and how this may influence coupled systems such as the calcium handling, energetic production, and protein expression systems in cells and tissue. In turn this should provide new insight into which levels of structural organization are most affected during the initiation and propagation of disease phenotype, and whether the disease phenotype requires neuro-humeral input.

A blessing and a curse central to the use of hiPSC-CMs as models for familial cardiomyopathies is the maturity of the cells. By most electrophysiological, morphological, metabolic, and mechanical measures, these cells are far from an adult phenotype (Yang et al., 2014). Although it can be difficult to match hiPSC-CMs to an exact gestational age *in vivo*, a recent study suggests when cells are matured on nanopatterned surfaces for out to 80–100 days in culture they have adult-like cell size and morphological characteristics. These cells express the adult form of cardiac myosin (β-myosin from MYH7), although isoform studies of troponin I suggest a fetal stage of expression (Bedada et al., 2014). Additionally, their myofibril mechanical properties and sarcomere ultrastructure match those of myofibrils from 75 day fetal heart tissue (Pioner et al., 2016). Thus, caution should be used in judging the developmental state of these hiPSC-CMs, based on appearance at the light microscope level.

Phenotyping cells at such an immature state is also a double-edged sword for studying genetic cardiomyopathies (summarized in Table 1). Many genetic cardiomyopathies clinically present in adulthood, and there are concerns about whether *in vitro* modeling will demonstrate an appropriate disease phenotype that recapitulates what occurs *in vivo*. However, in these familial-based diseases there are likely changes occurring in the myocardium well before many cardiomyopathies are detected clinically that can be identified using *in vitro* assays. For example, a recent study using hiPSC-CMs to study titin truncating mutations as a basis for dilated cardiomyopathy noted a disease phenotype in several assay systems, despite the fact that DCM associated with these mutations is usually detected well into adulthood (Hinson et al., 2015).

**Table 1:** **A summary of the benefits and drawbacks of using hiPSC-CMs to study familial cardiomyopathies**.

**Pros**	**Cons**
Moderate throughput for studying different mutations using CRISPR/Cas9	Lack of neuro-hormonal, endocrine, and paracrine responses
Able to study mutations that would be lethal *in vivo*	Immaturity of protein expression, ultrastructure, morphology, mechanical properties
Provides insight during development of disease to understand mechanisms	
Using isogenic controls allows for rigorous tests of causality and comparisons of mutations	

In fact, the immaturity of hiPSC-CMs can be beneficial for studying genetic cardiomyopathies. Studies that utilize animal models of cardiomyopathy often focus on characterizing end-stage phenotypes, contributing data mostly to enhance understanding of the final presentation of the disease. Alternatively, the fetal-like properties of hiPSC-CMs can provide mechanistic insight into early differences in function and structure present in cardiomyopathy that may be difficult or impossible to tease out in an *in vivo* system. For mutations in proteins that are not expressed initially in cardiomyocyte differentiation, but later in the timeline of development, it should be possible to determine the seminal event that leads to disease development and associated compensatory mechanisms. This developmental view of disease progression is particularly important as genetic testing grows in prevalence and robustness, and the possibility of pre-emptively treating genetic diseases before they present clinically becomes more likely. Using hiPSC-CMs as a model of genetic cardiomyopathies can allow for these crucial studies to understand how they develop, so that the progression or development of them can be prevented.

Looking forward, additional new tools are being developed that, combined with current technology, will allow for more and better models of disease in a dish. One example of this is the development of hiPSC lines that express proteins from the endogenous loci with a linked fluoro-tag that allow for studies of structural development with repeated live cell imaging (Ding et al., 2013). Another tool is optogenetics, which has been used primarily by neuroscientists to date (Steinbeck et al., 2015). The ability to express channels that can provide temporal and spatial control of stimulation or inhibition of depolarization could be quite useful for developing culture-based models of arrhythmia-genesis and long Q-T syndrome, but may also provide insight to how sarcomere protein mutations disrupt the balance between the contractile apparatus and Ca2+ cycling dynamics.

There still remain many limitations of *in vitro* models and some aspects of disease will be challenging or impossible to study using hiPSC-CMs. Examples include the complex temporal and spatial influence of neuro-hormonal, paracrine, and endocrine factors and how these are influenced by the structural and functional changes resulting from mutant protein expression. On the other hand, these new and emerging approaches will allow for studies that are impossible to perform with other platforms (e.g., early development, studying mutations that would be lethal) and inform on design of animal models that can probe those aspects. Ultimately these tools will be most useful when used in conjunction with *in vivo* models.

In summary, a new generation of biophysical, gene editing, and bioengineering approaches that are emerging holds great promise and potential as tools to improve our understanding of cardiac muscle development and the initiation and early-stage progression of disease development. These, in turn, will allow for better models used for drug and small molecule screening and the next generation of targeted therapies for heart failure.

## Author contributions

RZ and MR contributed equally in the ideas and writing of the manuscript.

### Conflict of interest statement

The authors declare that the research was conducted in the absence of any commercial or financial relationships that could be construed as a potential conflict of interest.
